# Need for evidence on long-term prognosis of PD+HD: a commentary

**DOI:** 10.1186/s12882-020-02212-x

**Published:** 2021-01-07

**Authors:** Mototsugu Tanaka, Naobumi Mise

**Affiliations:** 1 Division of Nephrology and Endocrinology, The University of Tokyo School of Medicine, 7-3-1 Hongo, Bunkyo-ku, Tokyo 113-8655, Japan; 2 Department of Nephrology, Division of Internal Medicine, Mitsui Memorial Hospital, 1 Kanda-izumi-cho, Chiyoda-ku, Tokyo 101-8643, Japan

**Keywords:** Peritoneal dialysis, Hemodialysis, Combined dialysis, End-stage kidney disease, End-stage renal disease, Residual kidney function, Technique failure, Precision medicine

## Abstract

Combination therapy with peritoneal dialysis and hemodialysis (PD+HD) is an alternative dialysis method for patients with end-stage kidney disease (ESKD). The complementary use of once-weekly HD expedites to achieve adequate dialysis and enables to prolong PD duration. Although PD+HD has been widely employed among Japanese PD patients, it is much less common outside Japan. Clinical evidences are still not enough, especially in long-term prognosis and appropriate treatment duration, suitable patients, and generalizability. A retrospective cohort study by Chung et al. (BMC Nephrol 21:348, 2020) compared the risk of mortality and hospitalization between PD patients who were transferred to PD+HD and those who were transferred to HD in Taiwan. Because the mortality and hospitalization rates did not differ between the groups, the authors concluded that, PD+HD may be a rational and cost-effective treatment option. It should be noted that the effects of PD+HD on long-term prognosis are still unknown due to too-short PD+HD duration. However, the study identified the high-risk patient population and showed the generalizability of PD+HD. PD+HD is a treatment of choice in patients with ESKD who prefer PD lifestyles even after decline in residual kidney function.

## Background

**Fig. 1:**
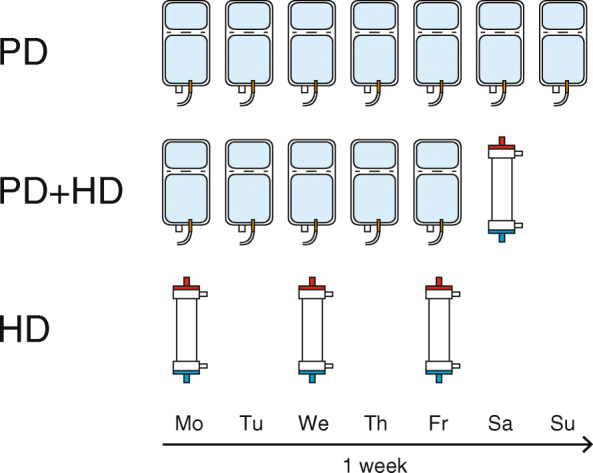
Treatment patterns of each dialysis modality. Shown are examples of treatment schedule in a week among PD, PD+HD, and HD. For each modality, the frequency of treatment can be modified based on patient characteristics. PD+HD may be used as either a bridging therapy or a maintenance therapy. Complementary use of HD increases dialysis doses and fluid removal in PD patients after decline in residual kidney function. PD+HD may also allow “PD-holiday” that mitigates excessive exposure to glucose dialysate. Abbreviations: HD, hemodialysis; PD, peritoneal dialysis; PD+HD, combination therapy with peritoneal dialysis and hemodialysis

Combination therapy with peritoneal dialysis and hemodialysis (PD+HD) has been widely used in Japan (Fig. 1). Currently, one-fifth of Japanese PD patients are on PD+HD, and 87.9% of those are treated with 5 to 6 days of PD and once-weekly HD (Japanese Society for Dialysis Therapy, unpublished data), which is covered by the national health insurance as a maintenance dialysis. Since the complementary use of HD ameliorates underdialysis and overhydration and enables significant prolongation of PD, PD+HD is preferred by PD patients who wish to maintain PD lifestyles after decline in residual kidney function [1, 2]. In fact, health-related quality of life for PD+HD was close to that for PD but was better than HD in role and social functions [3]. In this cohort, hospitalization risk was similar between PD+HD and HD, although PD+HD may have a higher hospitalization risk of dialysis access-related complications than HD [4]. However, many of these findings were from low quality studies, and several important arguments, such as long-term prognosis and appropriate treatment duration, and suitable patients, have not been determined. The generalizability of PD+HD was also unclear, since there have been few reports from outside Japan so far.

## PD+HD compared to HD

Chung et al. recently reported a retrospective cohort study comparing the risk of mortality and hospitalization (including emergent hospital visits) between PD+HD group (transfer from PD to PD+HD) and HD group (transfer from PD to HD) using a health insurance database in Taiwan [5]. An intention-to-treat analysis revealed that both risks were similar between the two groups, although peritonitis was a strong risk factor for hospitalization in PD+HD. The authors concluded that PD+HD is a safe, rational, and may be a cost-effective treatment for patients with end-stage kidney disease (ESKD), and the larger number of patients and longer observation period than previous studies may strengthen the present study conclusion.

However, long-term prognostic effects of PD+HD have not been clarified yet. In the present study, about 58% of patients were transferred from PD+HD to HD within a year. Too-short PD+HD duration makes it difficult to understand the crude effects of the combined dialysis in a 12-year follow-up. In addition, it remains unclear whether patients with ESKD can be treated adequately with PD+HD for a long period. A multivariate analysis including PD+HD duration may help to understand the long-term prognostic impacts and appropriate treatment period of this combined modality.

Nevertheless, the present study provided an important finding that PD+HD had a similar prognosis to HD, which was compatible with a recent Japanese study [4]. The consistency of the results from different regions suggested the feasibility and generalizability of PD+HD. There may be some differences in health insurance policies and the treatment strategies for additional HD between Japan and Taiwan. While once-weekly HD is routinely performed in most Japanese PD+HD patients, the study showed that a half of PD+HD patients in Taiwan were treated with only two HD sessions per month, and in such patients, HD was sometimes used as a rescue treatment. This biweekly HD regimen of PD+HD may increase the generalizability in developing countries.

The present study also suggested that PD+HD can be prescribed as an individualized, bridging dialysis modality. The flexibility may enable a smooth and appropriate transition of dialysis modality, and it may be of help during disastrous situations, such as recent COVID-19 pandemic [6]. On the other hand, dialysis access-related complications [4] and peritonitis [5] were risk factors of hospitalization for PD+HD, and technique survival rate was poor in those who required high ultrafiltration volume by additional HD [7]. It is necessary to identify suitable patients and establish the optimal indication for PD+HD.

In the present study, the authors proposed PD+HD as a part of an integrated dialysis care and mentioned that dialysis staff should be familiar with this combined modality. A patient-centered dialysis prescription by the shared decision-making is desired [8], however, many patients are feeling that they had not been sufficiently explained about dialysis modalities at the start of dialysis [9]. An increased awareness of PD+HD would provide patients more treatment choices in the precision medicine era.

## Conclusions

A recent study by Chung et al. suggested that mortality and hospitalization risks were similar between PD patients who were transferred to PD+HD and those who were transferred to HD in Taiwan [5]. However, the effects of PD+HD on long-term prognosis was unclear, since the treatment duration of PD+HD was too-short in the study. Nevertheless, this interesting paper provided several important findings. Firstly, PD+HD may be a safe, feasible, and flexible dialysis modality which is generalizable both in Japan and Taiwan. Secondary, patients with recent peritonitis were at a high risk of hospitalization. Clinicians and nurses should have a good understanding of PD+HD to apply precision medicine in clinical practice.

## Data Availability

Not applicable.

## Abbreviations

PD+HD: Combination therapy with peritoneal dialysis and hemodialysis
ESKD: End-stage kidney disease
COVID-19: Coronavirus disease 2019

## References

[1] Kawanishi H, Hashimoto Y, Nakamoto H, Nakayama M, Tranaeus A (2006). Combination therapy with peritoneal dialysis and hemodialysis. Perit Dial Int.

[2] Kawanishi H, Moriishi M (2007). Clinical effects of combined therapy with peritoneal dialysis and hemodialysis. Perit Dial Int.

[3] Tanaka M, Ishibashi Y, Hamasaki Y, Kamijo Y, Idei M, Kawahara T, Nishi T, Takeda M, Nonaka H, Nangaku M (2020). Health-related quality of life on combination therapy with peritoneal dialysis and hemodialysis in comparison with hemodialysis and peritoneal dialysis: a cross-sectional study. Perit Dial Int.

[4] Tanaka M, Ishibashi Y, Hamasaki Y, Kamijo Y, Idei M, Kawahara T, Nishi T, Takeda M, Nonaka H, Nangaku M (2020). Hospitalization for Patients on Combination Therapy With Peritoneal Dialysis and Hemodialysis Compared With Hemodialysis. Kidney Int Rep.

[5] Chung MC, Yu TM, Wu MJ, Chuang YW, Muo CH, Chen CH, Chang CH, Shieh JJ, Hung PH, Chen JW (2020). Is combined peritoneal dialysis and hemodialysis redundant? A nationwide study from Taiwan. BMC Nephrol.

[6] Matsuo N, Yokoyama K, Tanno Y, Yamamoto I, Yokoo T (2015). Combined therapy using peritoneal dialysis and hemodialysis may increase the indications for peritoneal dialysis in the United States. Kidney Int.

[7] Tanaka M, Ishibashi Y, Hamasaki Y, Kamijo Y, Idei M, Nishi T, Takeda M, Nonaka H, Nangaku M, Mise N (2020). Ultrafiltration volume by once-weekly hemodialysis is a predictor of technique survival of combination therapy with peritoneal dialysis and hemodialysis. Ther Apher Dial.

[8] Blake PG, Brown EA (2020). Person-centered peritoneal dialysis prescription and the role of shared decision-making. Perit Dial Int.

[9] Dahlerus C, Quinn M, Messersmith E, Lachance L, Subramanian L, Perry E, Cole J, Zhao J, Lee C, McCall M (2016). Patient Perspectives on the Choice of Dialysis Modality: Results From the Empowering Patients on Choices for Renal Replacement Therapy (EPOCH-RRT) Study. Am J Kidney Dis.

